# Association Between Hyperuricemia and Acute Ischemic Stroke in Patients at a Tertiary Care Hospital

**DOI:** 10.7759/cureus.10899

**Published:** 2020-10-11

**Authors:** Muhammad Irfan, Wajid Jawaid, Owais Hashmat, Qamar Nisa, Dipanty R Khastoori, Naila N Shahbaz

**Affiliations:** 1 Neurology, Dow University of Health Sciences, Karachi, PAK; 2 Neurology, Aga Khan University, Karachi, PAK

**Keywords:** ischemic stroke, cases, controls, hyperuricemia, hypertension, diabetes mellitus, dyslipidemia

## Abstract

Introduction

Stroke is the most debilitating of neurologic diseases. The rationale of the current study was to determine the association between hyperuricemia and ischemic stroke to establish a local perspective.

Methods

A total of 148 patients at a tertiary care hospital in Pakistan who fulfilled the inclusion criteria were enrolled in the study and then equally distributed into two study groups consisting of cases and controls (n = 74 in each group). In this study, there were 36 (48.6%) participants in the case group with hyperuricemia and ischemic stroke and 18 (24.3%) participants in the control group with hyperuricemia. The mean and standard deviations were computed for quantitative variables such as age, body mass index (BMI), and duration of stroke. Frequencies and percentages for the qualitative variables such as gender, hypertension, type 2 diabetes (T2D), dyslipidemia, smoking status, socioeconomic status, educational level, and hyperuricemia were calculated. The chi-square test was applied to compare both groups, with p ≤ 0.05 indicating significance. The odds ratio was also calculated to determine the association between case and control. Effect modifiers were controlled through stratification of age, gender, BMI, duration of stroke, hypertension, T2D, dyslipidemia, socioeconomic status, educational level, and smoking status to determine the effect of these on outcome variables. A post-stratification chi-square test was applied, with p ≤ 0.05 indicating statistical significance.

Results

In our study, stratification of hyperuricemia into cases and controls was performed for age, gender, T2D, hypertension, dyslipidemia, smoking status, socioeconomic status, and educational status. The maximum results were significant, with high strength of association between both groups. In the case group, the frequency of elevated uric acid was significantly higher than that of the control group. A comparison of hyperuricemia indicated p = 0.002, with an odds ratio of 2.95, which showed that elevated uric acid could be taken as a predictor of ischemic stroke. The uric acid level was significantly higher in men than in women. Additionally, hyperuricemia was associated with dyslipidemia. In patients with ischemic stroke, there was a significant association between serum uric acid level and T2D, hypertension, and smoking.

Conclusions

This study showed that the prevalence of hyperuricemia in patients with ischemic stroke was significantly higher as compared to the healthy population. Hyperuricemia can be considered as a risk factor for ischemic stroke because of its high prevalence in ischemic stroke patients. Our study explored the relationship between stroke and hyperuricemia and enabled increased understanding for caregivers so that effective management plans can be formulated for patients with ischemic stroke to prevent adverse outcomes.

## Introduction

Stroke is one of the most debilitating neurologic diseases and is the second most common cause of death and the third leading cause of disability in developed and developing countries [1]. According to a study, approximately 13.7 million new cases of stroke occur each year [2]. No large-scale epidemiological studies are available to determine the true incidence of stroke in Pakistan. The estimated annual incidence is 250/100,000, translating to 350,000 new cases every year [3]. Commonly quoted fatality rates are 17% at six months and 22% at one year. Many risk factors are involved in the development of stroke, including hypertension, cigarette smoking, hyperlipidemia, and diabetes [4].

Serum uric acid is considered as a risk marker for ischemic stroke. It is associated with endothelial dysfunction, coronary artery disease, and peripheral arterial disease, as well as nonalcoholic fatty liver disease [5]. Hyperuricemia has been linked with macrovascular and microvascular diseases in type 2 diabetes (T2D) patients. However, high serum uric acid level as an independent risk factor for cardiovascular disease, including stroke, is controversial [6]. Uric acid is the end-product of purine metabolism. It is a physiological free radical scavenger and one of the significant contributors of plasma antioxidant capacity. It plays a dual role, both as a prooxidant and as an antioxidant. Urate, the soluble form of uric acid, can scavenge the superoxide and the hydroxyl radicals and can chelate the transition metals [7]. In a study on the American population with acute stroke, those with higher serum levels of uric acid were more debilitated, with more recurrences and cardiovascular accidents [8]. A three-month follow-up of stroke patients in England indicated greater mortality for those with higher uric acid levels [9]. In one study conducted on patients of non-embolic ischemic stroke, higher uric acid levels resulted in enhanced risk of recurrence of stroke and associated complications [10]. Increased risks of both cerebrovascular and cardiovascular events were associated with hyperuricemia [11].

Some studies suggest antioxidant properties of uric acid offering neuroprotective role [12]. Similarly, one study noted lower mortality as well as complications in animal models with administration of uric acid [13]. Interestingly, less intense stroke lesions were noted in patients with higher uric acid levels [14]. Khalil et al. found that 23.3% of the patients with hyperuricemia had ischemic stroke in the case group and 6.7% of patients in the control group had hyperuricemia and no stroke [15].

Because of the ambiguity in previous studies on hyperuricemia, it is difficult to determine whether high uric acid levels are beneficial or detrimental or whether they can be used as a risk marker for ischemic stroke. Thus, we performed a retrospective study using hospital patients in Pakistan to determine the association between hyperuricemia and ischemic stroke.

## Materials and methods

We conducted a six-month case-control study of consenting stroke patients treated at the Department of Neurology in Civil Hospital, Karachi, Pakistan. Inclusion criteria included all patients of either sex aged 30 to 60 years admitted with ischemic stroke confirmed by a brain computed tomography (CT) scan showing a hypodense area. Patients who did not consent to be included were excluded from the study along with those with body mass index (BMI) > 30 kg/m^2^, a history of alcohol consumption, history of hypothyroidism or hyperthyroidism, myeloproliferative or lymphoproliferative disorders, renal impairment, chronic obstructive pulmonary disease, chronic liver disease, or congestive cardiac failure. Patients on medications such as thiazide or aspirin were also excluded. Patients with acute ischemic stroke were designated as case-patients and those without were labeled as control-patients. A brief history of the duration of illness and demographic information was taken from each patient and confirmed by an attendant. Blood samples were collected after 12-hour overnight fasting in a sterile manner for serum uric acid levels by our team on the first day of admission. The collected samples were sent to the same laboratory for biochemical analysis. All findings were entered into a proforma.

Data were analyzed using SPSS for Windows, Version 16.0 (SPSS Inc., Chicago, IL, USA). Mean and standard deviations were calculated for the quantitative variables such as age, BMI, and duration of stroke. Frequencies and percentages for the qualitative variables such as sex, hypertension, T2D, dyslipidemia, smoking status, socioeconomic status, educational level, and hyperuricemia were calculated.

Patients were considered as having ischemic stroke if they met two or more of the following criteria: Glasgow coma scale score < 15, inability to move one or more limbs, and a CT scan showing a hypodense area. We defined hyperuricemia as uric acid levels > 7 mg/dL for male patients or >6 mg/dL for female patients. Hypertension was defined as patients with systolic blood pressure > 140 mmHg or diastolic blood pressure > 90 mmHg and treated for more than five years. Patients with T2D had glycated hemoglobin > 6.5%. Patients with any one of the following were considered as having dyslipidemia: total cholesterol > 200 mg/dL, serum triglyceride levels > 150 mg/dL, low-density lipoprotein (LDL) > 100 mg/dL, or high-density lipoprotein (HDL) < 40 mg/dL. Patients who smoke at least five cigarettes per day for at least one year were deemed as smokers, and patients who had quit smoking for at least one year after being a smoker or those who had never smoked were deemed as non-smokers.

We applied the chi-square test to compare both groups, and a p-value of ≤0.05 was considered significant. We also calculated the odds ratio to see the association between case and control patients. Effect modifiers were controlled by stratifying age, gender, BMI, duration of stroke, hypertension, T2D, dyslipidemia, socioeconomic status, educational level, and smoking status to assess their impact on the outcome variables. We applied a post-stratification Chi-square test and considered a p-value of ≤0.05 as statistically significant. For sample size calculation, we used OpenEpi with alpha = 5%, power of the test 1-beta = 80, anticipated population proportion 1 = 23.3%, and anticipated population proportion 2 = 6.7%. The calculated sample size was a total of 148 patients, with 74 patients in each group. We applied non-probability consecutive sampling.

## Results

A total of 148 patients who fulfilled the inclusion criteria were enrolled in the study and were then equally distributed into two study groups. Table 1 shows that patients who were included in the study had an age range of 30 to 60 years, and the mean age of patients in the case group was 46.4 years (standard deviation [SD]: 9.0 years). The mean age of patients in the control group was 45.7 years (SD: 9.3 years). Table 2 shows the age group distribution, which was similar for each group. There were slightly more male patients than female patients in both groups (Table 3).

**Table 1:** Descriptive statistics of quantitative variables (n = 148) Abbreviation: BMI, body mass index

	n	Minimum	Maximum	Mean	Standard Deviation
Age (years)					
Case	74	30	60	46.4	9.04
Control	74	30	60	45.7	9.36
BMI (kg/m^2^)					
Case	74	22.5	29.6	24.2	3.7
Control	74	21.5	27	23.9	4.6
Duration of stroke (in hours)					
Case	74	3	12	4.5	2.6
Control	74	-	-	-	-

**Table 2:** Age distribution (n = 148)

Groups	Age (years)	Frequency	Percentage
Case	30-50	46	62.2%
	51-60	28	38.8%
	Total	74	100.0
Control	30-50	46	62.2%
	51-60	28	38.8%
	Total	74	100.0

**Table 3:** Gender distribution (n = 148)

Groups	Gender	Frequency	Percentage
Case	Female	33	44.6
	Male	41	55.4
	Total	74	100.0
Control	Female	30	40.5
	Male	44	59.5
	Total	74	100.0

Most patients (71.6%; n = 53) in the case group had T2D, whereas a smaller majority of patients in the control group (51.4%, n = 38) had T2D (Table 4). Hypertension was more common in the case group (64.9%; n = 48) than the control group (Table 5). Table 6 presents the distribution of smoking status. Dyslipidemia was more prevalent in the case group than in the control group (Table 7). Table 8 presents the socioeconomic status distribution, and Table 9 shows the distribution of educational status. In Table 10, the distribution of hyperuricemia showed that 36 (48.6%) participants had hyperuricemia in the case group and 18 (24.3%) participants had hyperuricemia in the control group, indicating that the frequency of elevated uric acid was significantly higher as compared to the control group (p = 0.002; odds ratio: 2.95).

**Table 4:** Distribution of T2D (n = 148) Abbreviation: T2D, type 2 diabetes

Groups	T2D	Frequency	Percentage
Case	Yes	53	71.6%
	No	21	28.4%
	Total	74	100%
Control	Yes	38	51.4%
	No	36	48.6%
	Total	74	100%

**Table 5:** Distribution of hypertension (n = 148)

Groups	Hypertension	Frequency	Percentage
Case	Yes	48	64.9%
	No	26	35.1%
	Total	74	100%
Control	Yes	30	40.54%
	No	44	59.46%
	Total	74	100%

**Table 6:** Distribution of smoking (n = 148)

Groups	Smoking	Frequency	Percentage
Case	Yes	40	54.1%
	No	34	45.9%
	Total	74	100%
Control	Yes	31	41.9%
	No	43	58.1%
	Total	74	100%

**Table 7:** Distribution of dyslipidemia (n = 148)

Groups	Dyslipidemia	Frequency	Percentage
Case	Yes	45	60.8%
	No	29	39.2%
	Total	74	100%
Control	Yes	35	47.3%
	No	39	52.7%
	Total	74	100%

**Table 8:** Distribution of socioeconomic status (n = 148)

Groups	Socioeconomic Status	Frequency	Percentage
Case	Lower class	20	27.02%
	Middle class	34	45.94%
	Upper class	20	27.02%
Control	Lower class	12	16.21%
	Middle class	22	29.72%
	Upper class	40	54.05%

**Table 9:** Distribution of educational status (n = 148)

Groups	Educational Status	Frequency	Percentage
Case	Illiterate	3	4.05%
	Primary	10	13.51%
	Secondary	15	20.27%
	Higher	46	62.16%
Control	Illiterate	5	6.75%
	Primary	13	17.56%
	Secondary	21	28.37%
	Higher	35	47.29%

**Table 10:** Distribution and comparison of hyperuricemia in both groups (n = 148)

Groups	Hyperuricemia	Frequency	Percentage
Case	No	38	51.4
	Yes	36	48.6
	Total	74	100.0
Control	No	56	75.7
	Yes	18	24.3
	Total	74	100.0

Tables 11-18 show the stratification of hyperuricemia in cases and controls with respect to age, gender, T2D, hypertension, dyslipidemia, smoking status, socioeconomic status, and educational status. The maximum results were significant and exhibited high strength of association between both of the groups.

**Table 11:** Stratification of hyperuricemia in both groups with regard to age (n = 148) Values were obtained by applying the chi-square test. p-Value = 0.002. OR = 2.95. Abbreviation: OR, odds ratio

Age			Hyperuricemia		Total	p-Value, OR
			Yes	No		
30-50 years	Group	Case	20	20	40	0.0083, 3.333
		Control	12	40	52	
	Total	32	60	92		
51-60 years	Group	Case	16	18	34	0.143, 2.370
		Control	6	16	22	
	Total	22	34	56		
Total	Group	Case	36	38	74	0.002, 2.95
		Control	18	56	74	
	Total	54	94	148		

**Table 12:** Stratification of hyperuricemia in both groups with regard to gender (n = 148) Abbreviation: OR, odds ratio

Gender			Hyperuricemia		Total	p-Value, OR
			Yes	No		
Male	Group	Case	22	16	38	0.008, 5.09
		Control	10	37	47	
	Total	32	53	85		
Female	Group	Case	14	22	36	0.446, 1.511
		Control	8	19	27	
	Total	22	41	63		
Total	Group	Case	36	38	74	0.002, 2.95
		Control	18	56	74	
	Total	54	94	148		

**Table 13:** Stratification of hyperuricemia in both groups with regard to T2D (n = 148) Abbreviations: T2D, type 2 diabetes; OR, odds ratio

T2D			Hyperuricemia		Total	p-Value, OR
			Yes	No		
Yes	Group	Case	21	15	36	0.003, 3.73
		Control	15	40	55	
	Total	36	55	91		
No	Group	Case	15	23	38	0.079, 3.478
		Control	3	16	19	
	Total	18	39	57		
Total	Group	Case	36	38	74	0.002, 2.95
		Control	18	56	74	
	Total	54	94	148		

**Table 14:** Stratification of hyperuricemia in both groups with regard to hypertension (n = 148) Abbreviation: OR, odds ratio

Hypertension			Hyperuricemia		Total	p-Value, OR
			Yes	No		
Yes	Group	Case	22	13	35	0.006, 5.58
		Control	10	33	43	
	Total	32	46	78		
No	Group	Case	14	25	39	0.36, 1.61
		Control	8	23	31	
	Total	22	48	70		
Total	Group	Case	36	38	74	0.002, 2.95
		Control	18	56	74	
	Total	54	78	148		

**Table 15:** Stratification of hyperuricemia in both groups with regard to smoking (n = 148) Abbreviation: OR, odds ratio

Smoking			Hyperuricemia		Total	p-Value, OR
			Yes	No		
Yes	Group	Case	26	19	45	0.52, 1.37
		Control	13	13	26	
	Total	39	32	71		
No	Group	Case	10	19	29	0.013, 4.526
		Control	5	43	48	
	Total	15	62	77		
Total	Group	Case	36	38	74	0.002, 2.95
		Control	18	56	74	
	Total	54	94	148		

**Table 16:** Stratification of hyperuricemia in both groups with regard to dyslipidemia (n = 148) Abbreviation: OR, odds ratio

Dyslipidemia			Hyperuricemia		Total	p-Value, OR
			Yes	No		
Yes	Group	Case	20	18	38	0.002, 4.72
		Control	8	34	42	
	Total	28	52	80		
No	Group	Case	16	20	36	0.27, 1.76
		Control	10	22	32	
	Total	26	42	68		
Total	Group	Case	36	38	74	0.002, 2.95
		Control	18	56	74	
	Total	54	94	148		

**Table 17:** Stratification of hyperuricemia in both groups with regard to socioeconomic status (n = 148) Abbreviation: OR, odds ratio.

Socioeconomic Status			Hyperuricemia		Total	p-Value, OR
			Yes	No		
Lower	Group	Case	10	8	18	0.13, 3.13
		Control	4	10	14	
	Total	14	18	32		
Middle	Group	Case	6	15	21	0.067, 5.00
		Control	2	25	33	
	Total	8	40	56		
Upper	Group	Case	20	15	35	0.48, 1.44
		Control	12	13	25	
	Total	32	28	60		
Total	Group	Case	36	38	74	0.002, 2.95
		Control	18	56	74	
	Total	54	94	148		

**Table 18:** Stratification of hyperuricemia in both groups with regard to educational status (n = 148) Abbreviation: OR, odds ratio

Educational Status			Hyperuricemia		Total	p-Value, OR
			Yes	No		
Illiterate	Group	Case	4	1	5	0.67, 2.00
		Control	2	1	3	
	Total	6	2	8		
Primary	Group	Case	11	5	16	0.09, 5.50
		Control	2	5	7	
	Total	13	10	23		
Secondary	Group	Case	12	12	24	0.001, 14.00
		Control	4	56	74	
	Total	32	78	36		
Higher	Group	Case	9	20	29	0.233, 1.89
		Control	10	42	52	
	Total	19	62	81		

Table 19 shows the stratification of hyperuricemia in cases and controls with respect to BMI, and the maximum results were significant and exhibited high strength of association between both of the groups (p = 0.002; odds ratio: 2.95). Table 20 shows stratification of hyperuricemia with regard to duration of stroke.

**Table 19:** Stratification of hyperuricemia in both groups with regard to BMI (n = 148) Abbreviations: BMI, body mass index; OR, odds ratio

BMI			Hyperuricemia		Total	p-Value, OR
			Yes	No		
<22 kg/m^2^	Group	Case	18	10	28	0.001, 7.2
		Control	9	36	45	
	Total	27	46	73		
>22 kg/m^2^	Group	Case	18	28	46	0.25, 1.86
		Control	9	26	35	
	Total	26	42	81		
Total	Group	Case	36	38	74	0.002, 2.95
		Control	18	56	74	
	Total	54	94	148		

**Table 20:** Stratification of hyperuricemia in both groups with regard to duration of stroke (n = 148) Abbreviations: OR, odds ratio; NA, not applicable

Duration of Stroke			Hyperuricemia		Total	p-Value, OR
			Yes	No		
<6 hours	Group	Case	9	5	14	NA
		Control	-	-	-	
	Total	-	-	-		
>6 hours	Group	Case	28	22	50	NA
		Control	-	-	-	
	Total	-	-	-	74	

## Discussion

In this study, the serum uric acid levels in patients with acute stroke were noted in relation to the healthy population. The mean serum uric acid level was 5.94 (±1.70) mg/dL, and approximately half of the patients were hyperuricemic. According to a large 10-year follow-up study, the prevalence of hyperuricemia in the United States is 20.1% [16]. Another large study in the Bangkok population showed that the prevalence of hyperuricemia is 24.4% [17], and a study from the island of Seychelles reported that the prevalence of hyperuricemia is 35.2% in men and 8.7% in women [18]. According to these studies, the prevalence of hyperuricemia was significantly higher in patients with acute stroke as compared to the healthy population.

Stroke is one of the main clinical manifestations of cardiovascular disease, and findings from studies investigating the relationship between uric acid and stroke have been inconsistent. Some studies reported a positive independent relationship between uric acid and stroke, whereas others demonstrated that there was no significant relationship between uric acid and stroke occurrence, as discussed below.

Bansal et al. studied 50 patients with ischemic thrombotic cerebrovascular disease. Hyperuricemia was found in 30% of the cases, and they concluded that elevated serum uric acid level might play a role in the causation of ischemic thrombotic cerebrovascular disease in general, and especially in patients younger than 40 years [19]. Kim et al. conducted a systematic review and meta-analysis of 16 prospective cohort studies, including 238,449 adults, to assess the association between hyperuricemia and risk of stroke incidence and mortality [5]. They found that hyperuricemia may modestly but significantly increase the risk of both stroke incidence and mortality.

According to the results of Milionis et al., elevated serum uric acid levels were associated with an increased risk of acute ischemic stroke in the elderly [10]. However, in the Syst-Eur trial that included patients with isolated systolic hypertension, no significant relationship was detected between serum uric acid levels and fatal and non-fatal strokes [20]. Moreover, in a middle-aged Japanese population, hyperuricemia was not associated with stroke mortality in 108,284 person-years on follow-up [21]. After Goldberg et al. studied middle-aged men and followed them for 20 years, they determined that there was not a significant correlation between serum uric acid and thromboembolic stroke [22]. Chamorro et al. assessed the prognostic significance of serum uric acid concentration in patients with acute ischemic stroke, and they reported that patients with T2D had a lower uric acid concentration than other patients and that uric acid concentration was inversely associated with fasting blood glucose [14].

In our study, there was no significant association between serum uric acid level and serum triglyceride, total cholesterol, HDL cholesterol, and LDL cholesterol, but hyperuricemia was associated with serum triglycerides and LDL cholesterol in some studies such as the one by Zavaroni et al. [23] who studied 957 young men and demonstrated that there was a significant positive correlation between serum uric acid levels, and levels of serum triglyceride, total cholesterol, and LDL cholesterol. Moreover, Chamorro et al. [14] reported the association between the serum uric acid level and the amount of serum triglyceride.

The mechanism of this strong association between serum uric acid levels and triglyceride levels is still poorly understood. Although some studies reported the role of genetic factors in the occurrence of gout and hypertriglyceridemia, most researchers noted that hyperuricemia and hypertriglyceridemia might reflect the patient's lifestyle as a part of metabolic syndrome [11].

Different studies assessed the uric acid levels exclusively in patients with ischemic stroke where there was a significant association between the uric acid level and the incidence of acute ischemic stroke [11,24]. According to the results of Bos et al., the hazard ratio of elevated serum uric acid levels in ischemic stroke was 1.77, and in hemorrhagic stroke, it was 1.68 [24].

The role of urate in ischemic stroke is poorly understood. Therefore, the role of uric acid as a risk factor for vascular disease and acute stroke is controversial. Because there is a lack of information, we decided to determine serum uric acid levels in patients with acute stroke and assess its relationship with cardiovascular risk factors. Previous studies have indicated that hyperuricemia predicts ischemic heart disease in nondiabetics [23,25]. However, the data relating the risk of hyperuricemia to cerebrovascular disease are limited. 

Overwhelming evidence suggests that hyperuricemia is linked to obesity, hypertension, reduced HDL cholesterol [26], hypertriglyceridemia, hyperinsulinemia, and reduced insulin sensitivity [27]. We also observed that this association and the presence of multiple risk factors are likely to explain a substantial part of the increased risk of stroke. However, even after extensive adjustment for cardiovascular risk factors, the serum uric acid remained an independent risk factor for stroke. 

Our study had a few limitations. Our study used a smaller sample size of admitted patients compared to other studies we discussed. We also used a relatively short duration and narrow age range (30 to 60 years). Future studies with larger populations, broader age ranges, and longer durations will help mitigate these limitations.

## Conclusions

Our study showed that the prevalence of hyperuricemia in patients with acute ischemic stroke was significantly higher as compared to the healthy population. In terms of gender distribution, uric acid levels were significantly higher in men than in women. Hyperuricemia was also associated with dyslipidemia, and in patients with acute ischemic stroke, there was a significant association between serum uric acid levels, and diabetes mellitus, hypertension, and smoking. Therefore, as determined in our study, due to the high prevalence of hyperuricemia in patients with ischemic stroke, it should be considered as a risk factor for acute ischemic stroke.
